# Complete Versus Lesion-Only Primary PCI: The Randomized Cardiovascular MR CvLPRIT Substudy

**DOI:** 10.1016/j.jacc.2015.09.099

**Published:** 2015-12-22

**Authors:** Gerry P. McCann, Jamal N. Khan, John P. Greenwood, Sheraz Nazir, Miles Dalby, Nick Curzen, Simon Hetherington, Damian J. Kelly, Daniel J. Blackman, Arne Ring, Charles Peebles, Joyce Wong, Thiagarajah Sasikaran, Marcus Flather, Howard Swanton, Anthony H. Gershlick

**Affiliations:** ∗ Department of Cardiovascular Sciences, University of Leicester and the National Institute of Health Research (NIHR) Leicester Cardiovascular Biomedical Research Unit, University Hospitals of Leicester National Health Service (NHS) Trust, Glenfield Hospital, Leicester, United Kingdom; † Multidisciplinary Cardiovascular Research Centre & Division of Cardiovascular and Diabetes Research, Leeds Institute of Cardiovascular and Metabolic Medicine (LICAMM), University of Leeds, Leeds, United Kingdom; ‡ Department of Cardiology, Royal Brompton and Harefield Foundation Trust, Harefield Hospital, Middlesex, United Kingdom, and the Cardiovascular Biomedical Research Unit of Royal Brompton and Harefield NHS Foundation Trust and Imperial College London, London, United Kingdom; § Department of Cardiology and Radiology, University Hospital Southampton NHS Foundation Trust and University of Southampton, Southampton, United Kingdom; ‖ Department of Cardiology, Kettering General Hospital, Kettering, United Kingdom; ¶ Department of Cardiology, Royal Derby Hospital, Derby, United Kingdom; # Leicester Clinical Trials Unit, University of Leicester, Leicester, United Kingdom; ∗∗ Department of Mathematical Statistics and Actuarial Science, University of the Free State, Bloemfontein, South Africa; †† Clinical Trials & Evaluation Unit, Royal Brompton & Harefield NHS Foundation Trust and Imperial Clinical Trials Unit, Imperial College London, London, United Kingdom; ‡‡ Clinical Trials Unit, Norfolk and Norwich University Hospitals NHS Foundation Trust and Norwich Medical School, University of East Anglia, Norwich, United Kingdom; §§ Department of Cardiology, Heart Hospital, University College London Hospitals, London, United Kingdom

**Keywords:** CMR, complete revascularization, multivessel disease, PPCI, STEMI, AAR, area at risk, CMR, cardiovascular magnetic resonance, IRA, infarct-related artery, LGE, late gadolinium-enhanced, LV, left ventricle/ventricular, MACE, major adverse cardiovascular events, MI, myocardial infarction, MSI, myocardial salvage index, MVO, microvascular obstruction, PCI, percutaneous coronary intervention, PPCI, primary percutaneous coronary intervention, STEMI, ST-segment elevation myocardial infarction, T2w-STIR, T2-weighted short tau inversion recovery, TIMI, Thrombolysis In Myocardial Infarction

## Abstract

**Background:**

Complete revascularization may improve outcomes compared with an infarct-related artery (IRA)-only strategy in patients being treated with primary percutaneous coronary intervention (PPCI) who have multivessel disease presenting with ST-segment elevation myocardial infarction (STEMI). However, there is concern that non-IRA PCI may cause additional non-IRA myocardial infarction (MI).

**Objectives:**

This study sought to determine whether in-hospital complete revascularization was associated with increased total infarct size compared with an IRA-only strategy.

**Methods:**

This multicenter prospective, randomized, open-label, blinded endpoint clinical trial evaluated STEMI patients with multivessel disease having PPCI within 12 h of symptom onset. Patients were randomized to either IRA-only PCI or complete in-hospital revascularization. Contrast-enhanced cardiovascular magnetic resonance (CMR) was performed following PPCI (median day 3) and stress CMR at 9 months. The pre-specified primary endpoint was infarct size on pre-discharge CMR. The study had 80% power to detect a 4% difference in infarct size with 100 patients per group.

**Results:**

Of the 296 patients in the main trial, 205 participated in the CMR substudy, and 203 patients (98 complete revascularization and 105 IRA-only) completed the pre-discharge CMR. The groups were well-matched. Total infarct size (median, interquartile range) was similar to IRA-only revascularization: 13.5% (6.2% to 21.9%) versus complete revascularization, 12.6% (7.2% to 22.6%) of left ventricular mass, p = 0.57 (95% confidence interval for difference in geometric means 0.82 to 1.41). The complete revascularization group had an increase in non-IRA MI on the pre-discharge CMR (22 of 98 vs. 11 of 105, p = 0.02). There was no difference in total infarct size or ischemic burden between treatment groups at follow-up CMR.

**Conclusions:**

Multivessel PCI in the setting of STEMI leads to a small increase in CMR-detected non-IRA MI, but total infarct size was not significantly different from an IRA-only revascularization strategy. (Complete Versus Lesion-Only Primary PCI Pilot Study [CvLPRIT]; ISRCTN70913605)

Multivessel coronary artery disease is seen in approximately 40% of patients presenting with ST-segment elevation myocardial infarction (STEMI) being treated with primary percutaneous coronary intervention (PPCI). Clinical guidelines recommend percutaneous coronary intervention (PCI) to the infarct-related artery (IRA) only, largely based on registry data that have suggested increased risk of adverse events with complete revascularization 1, 2 in those patients selected to receive complete revascularization. However, 2 recent prospective randomized controlled trials (PRAMI [Preventive Angioplasty in Myocardial Infarction] trial and the CvLPRIT [Complete Versus Lesion-Only Primary PCI Trial]), which compared a strategy of complete versus IRA-only revascularization in PPCI patients with multivessel disease, have shown a reduction in major adverse cardiovascular events (MACE) with complete revascularization 3, 4.

The mechanisms leading to improved clinical outcomes are currently unclear. However, there is concern that PCI to non-IRAs may be associated with additional procedural-related infarction (5). These well-described type 4a myocardial infarctions (MIs) (6) cannot be detected by conventional enzymatic markers at the time of PPCI because the associated increases are relatively small compared with the large rise in enzymes caused by the STEMI itself. Cardiovascular magnetic resonance (CMR) is able to precisely characterize areas of myocardial injury following myocardial ischemia. The myocardium at risk becomes edematous (7), and late gadolinium-enhanced (LGE) imaging allows the accurate detection and quantification of infarct size and microvascular obstruction (MVO) (8). Infarct size (9) and MVO (10) measured on CMR are both strong medium-term prognostic markers following PPCI. There are no CMR data as yet in the literature on patients undergoing complete revascularization for multivessel disease at the time of PPCI.

The primary aim of the current pre-specified substudy was to assess whether a complete revascularization strategy, due to causing additional infarcts in the non-IRA territories, was associated with greater infarct size than an IRA-only strategy in patients randomized in CvLPRIT. Additionally, we aimed to assess whether myocardial salvage and myocardial ischemia at follow-up CMR were different in the 2 groups.

## Methods

### Study design

The design and rationale of the study have been published previously (11). Briefly, CvLPRIT CMR was a pre-specified substudy of a multicenter, prospective, randomized, controlled, open-label clinical trial and with blinded CMR endpoint analysis (PROBE design) that was conducted in 7 U.K. centers between May 2011 and May 2014. The inclusion (PPCI <12 h from symptom onset and angiographic stenosis in the non-IRA >70% or >50% in 2 orthogonal views) and exclusion criteria were as for the main trial (4) with absolute contraindications to CMR imaging as an additional exclusion. The Trent Research Ethics Committee (Ref: 11/H0405/4) approved the study, which was conducted according to the Declaration of Helsinki. All patients gave written informed consent.

### Patients

**Figure 1:**
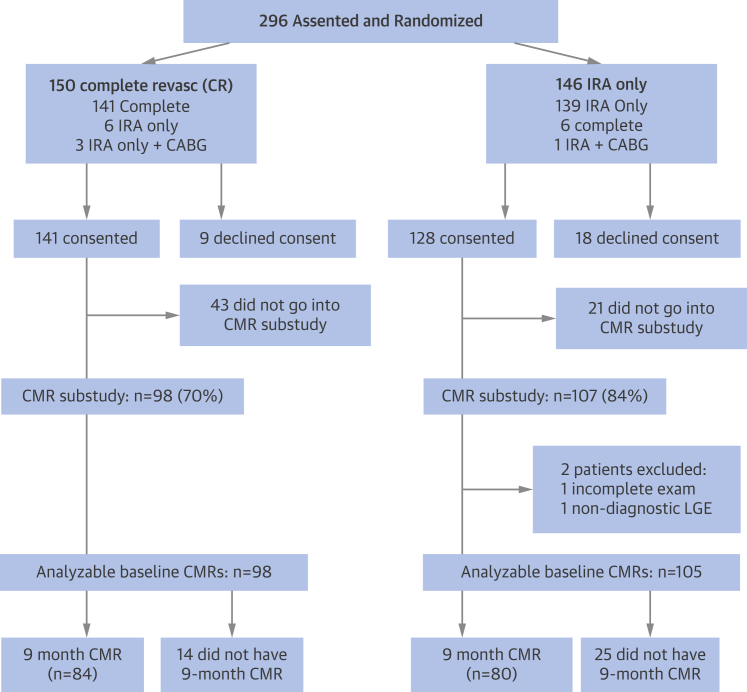
Consort Diagram for CvLPRIT CMR Standards of Reporting Trials (CONSORT) diagram illustrating recruitment and patient flow. In the **topmost boxes** are the numbers of patients randomized to each of the 2 treatment arms (intention to treat) and the number who subsequently received each treatment. CABG = coronary artery bypass graft; CMR = cardiovascular magnetic resonance; CR = complete revascularization; CvLPRIT = Complete Versus Lesion-Only Primary PCI Pilot Study; IRA = infarct-related artery; LGE = late gadolinium enhancement.

The flow diagram for patient recruitment and testing is shown in Figure 1. Eligible patients from the first 286 in the main trial (4) were approached to participate in the CMR substudy until the target recruitment (200) was achieved (April 2013). Patients in the CMR substudy had similar clinical characteristics to those included in the main trial (Table 1).

**Table 1:** Baseline Characteristics of the Main CvLPRIT and CMR Substudy Participants

	CvLPRIT (n = 296)	CMR Substudy		p Value
		CR (n = 98)	IRA (n = 105)	
Age, yrs	64.9 ± 11.6	63.1 ± 11.3	64.1 ± 10.8	0.53
Male	240/296 (81.1)	87 (88.8)	83 (79.0)	0.06
BMI, kg/m^2^∗	27.3 (24.4–30.2)	27.5 (24.6–29.7)	27.5 (24.7–30.6)	0.36
Systolic BP, mm Hg	137.6 ± 27.1	134.7 ± 27.3	140.0 ± 28.0	0.18
Anterior infarct	106 (35.6)	35 (35.7)	37 (37.2)	0.94
eGFR, ml/min/1.73	95.74 ± 34.7	98.2 ± 34.3	93.49 ± 30.7	0.36
Peak CK, IU/l∗	1,010 (423.3–1,740)	1,025 (628–1,660)	1,057 (614–1,834)	0.37
Hypertension	105/287 (36.6)	36 (36.7)	37 (35.2)	0.82
Hypercholesterolemia	75/287 (26.1)	28 (28.6)	28 (26.7)	0.76
Diabetes mellitus	39/287 (13.6)	15 (15.3)	13 (12.4)	0.55
Current smoker	87/285 (30.5)	36 (36.7)	28 (28.0)	0.12
Previous MI	12/287 (4.2)	4 (4.1)	4 (3.8)	0.92
Previous PCI	9/287 (3.1)	4 (4.1)	3 (2.9)	0.63
Killip class II–III	24/286 (8.4)	6 (6.1)	10 (9.5)	0.37

Values are mean ± SD, n/N (%), n (%), or median (interquartile range), unless otherwise as noted.

BME = black or minority ethnicity; BMI = body mass index; BP = blood pressure; CK = creatine kinase; CMR = cardiovascular magnetic resonance; CR = complete revascularization; CvLPRIT = Complete Versus Lesion-Only Primary PCI Pilot Study; eGFR = estimated glomerular filtration rate; IRA = infarct-related artery–only revascularization; MI = myocardial infarction; PCI = percutaneous coronary intervention.

∗ Non-normally distributed data: analyzed after log transformation with independent Student *t* testing.

### Randomization and treatment

Eligible patients presenting with STEMI within 12 h were randomized, after verbal assent and coronary angiography, but before PCI to the culprit lesion, to either IRA-only or in-hospital complete revascularization. Randomization was stratified by infarct location (anterior/non-anterior MI) and time to presentation (>3 or ≤3 h). PCI was performed according to current guidelines. Written informed consent for continued participation in the study was obtained on the day(s) following the PPCI, once the patient was able to understand and retain the information.

### Angiographic analysis

Pre- and post-PPCI epicardial coronary flow was assessed using Thrombolysis In Myocardial Infarction (TIMI) scoring (12). Collateral flow to the IRA pre-PPCI was graded using the Rentrop system (13). Quantitative coronary angiography was undertaken using QAngioXA v1.0 software (Medis, Leiden, the Netherlands).

### CMR imaging

CMR was undertaken in 5 of the 7 hospitals recruiting to the main study, using 1.5-T platforms (4 Siemens Avanto, Erlangen, Germany, and 1 Philips Intera, Best, the Netherlands). Patients from the 2 other participating hospitals without onsite CMR (Derby and Kettering) were scanned at Glenfield Hospital.

### Pre-discharge CMR

**Figure 2:**
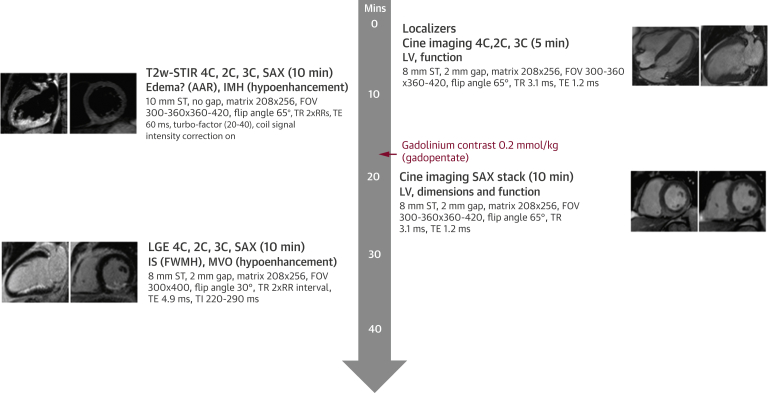
Pre-Discharge CMR Protocol Pulse sequence parameters for Siemens scanners given. 4/3/2C = 4/3/2-chamber long-axis; AAR = area at risk; CMR = cardiovascular magnetic resonance; FWHM = full-width half-maximum; FOV = field of view; IMH = intramyocardial hemorrhage; IS = infarct size; LGE = late gadolinium-enhanced; LV = left ventricular; MVO = microvascular obstruction; SAX = short-axis; ST = slice thickness; T2w-STIR = T2-weighted short tau inversion recovery; TE = echo time; TI = inversion time; TR = repetition time.

CMR was performed during the index admission and after non-IRA PCI in those patients in the complete revascularization group in whom the procedure was staged. The protocol was similar to that previously described (14) with the addition of T2-weighted short tau inversion recovery (T2w-STIR) imaging for the detection of edema and is shown in Figure 2, with typical pulse sequence parameters for the Siemens scanners. A complete T2w-STIR left ventricular (LV) short-axis stack was acquired after localizer and long-axis cine imaging. Gadolinium gadopentate (Magnevist, Bayer, Faversham, United Kingdom) 0.2 mmol/kg was administered before the short-axis cine stack.

### Follow-up CMR

Follow-up CMR was performed at 9 months (±4 weeks) post-PPCI. The protocol for follow-up CMR was similar to the pre-discharge scan, but with T2w-STIR imaging omitted and assessment of reversible ischemia included. First-pass perfusion imaging in 3 short-axis slices was performed as previously described (15) following intravenous administration of 0.1 mmol/kg gadolinium contrast, using a breath-hold, saturation recovery gradient-echo pulse sequence. Pharmacological stress was achieved with intravenous adenosine infusion at 140 μg/kg/min for ≥3 min. Rest perfusion, with a further 0.1 mmol/kg of contrast, was performed after acquiring a short-axis cine stack covering the entire LV and ≥10 min after stress imaging. LGE imaging was acquired 10 min following rest perfusion.

### CMR analysis

Physicians blinded to all clinical data, including treatment allocation, performed the CMR analyses at the University of Leicester core laboratory. Image quality was assessed on a 4-point scale: 3 = excellent; 2 = good; 1 = moderate; and 0 = unanalyzable. Additionally, for T2w-STIR sequences, if no regional variation in signal intensity within the myocardium was seen, these patients were excluded from analysis of the area at risk (AAR).

LV volumes and mass were calculated from cine images as previously described using QMass v7.1 (Medis) (15). The presence of LGE was assessed by 2 observers (G.P.M., J.N.K.) and was quantitated with cvi42 (Cardiovascular Imaging, Calgary, Alberta, Canada) using the full-width half-maximum technique (16). If infarction was seen in more than 1 coronary territory in the pre-discharge CMR, this was recorded as being in the IRA territory (associated edema and/or MVO) or the non-IRA territory with the consensus of 3 observers (J.N.K., G.P.M., J.P.G.). Non-IRA infarcts were additionally classified as likely to be acute or chronic (presence of wall thinning and no edema/MVO). Infarct size was recorded for both IRA and non-IRA LGE, and total infarct size was the sum of all LGE. Edema (AAR) was quantified as hyperenhancement on T2w-STIR imaging in cmr42 using Otsu’s Automated Method (17). Areas of hypoenhancement within infarct and edema were regarded as MVO and intramyocardial hemorrhage, respectively, and included in the infarct size and AAR, respectively. LV volumes and mass were indexed to body surface area, and infarct size was expressed as percentage of LV mass. Myocardial salvage index (MSI) was calculated as the percentage of the AAR that was not infarcted on LGE images using infarct size from both the pre-discharge (Acute MSI) and follow-up (Final MSI) CMR scan.

Perfusion images were visually assessed for defects (visible defect for ≥5 heartbeats) by the consensus of 2 observers (J.N.K., G.P.M.). Cine, stress perfusion, rest perfusion, and LGE images were studied together and assessed according to the American Heart Association 16-segment model. Rest perfusion images were used mainly to identify artifacts. Perfusion defects and areas of infarction were graded as subendocardial (≤50% transmurality) or transmural (>50% transmurality) and given a score of 1 or 2, respectively, per segment, whereas normal myocardium was scored 0. A modified summed difference score was calculated (maximum score 32) (18), defined as the difference between the sum of segmental stress perfusion defects and LGE. The summed difference score was expressed as percentage of the maximum possible to calculate ischemic burden.

### Intra- and interobserver variability of LV volumetrics and infarct characteristics

Ten pre-discharge and follow-up scans were randomly selected and analyzed twice by the same observer after 4 weeks (J.N.K.) and once by a further observer (S.N.). The data are shown in the Online Appendix. All intraclass correlation coefficients for intraobserver and interobserver agreement for CMR quantitative data exceeded 0.92.

### Clinical outcomes and follow-up

MACE comprised a composite of all-cause mortality, recurrent MI, heart failure, and ischemia-driven revascularization. Additional secondary endpoints included cardiovascular death, individual components of the primary endpoint, and the safety endpoints stroke, major bleeding, and contrast-induced nephropathy. Data were collected by an independent clinical trials unit (Royal Brompton Hospital, London, England) and events adjudicated by blinded clinicians.

### Statistical analysis

The primary outcome was infarct size (expressed as a % of LV mass) on pre-discharge CMR, which was analyzed on a log-transformed scale, as it is generally right-skewed. Primary analysis was on an intention-to-treat basis of all randomized patients according to treatment group who completed the pre-discharge CMR. The result was adjusted for known predictors of infarct size (age, anterior MI, time to revascularization, diabetes, AAR, Rentrop grade, and TIMI flow grade pre-PPCI), using generalized mixed models. No adjustments for multiplicity were performed for secondary endpoints. Normally distributed continuous variables were expressed as mean ± SD, and comparison was with Student *t* tests. Non-normally distributed data were expressed as median (25th to 75th quartiles) and analyzed using independent Student *t* testing where log transformation normalized data, and using Mann-Whitney testing were the degree of skew rendered data nontransformable. Categorical variables were compared using chi-square testing. Clinical outcomes were assessed using time-to-first-event survival analysis (log-rank test with right censoring), and Cox proportional hazard models were fitted to estimate hazard ratios and 95% confidence intervals for treatment comparisons. One hundred patients in each arm gave 81% power to detect a 4% absolute difference in infarct size, assuming a mean of 20% of LV mass and standard deviation of 10% 19, 20, using a 2-tailed test with alpha = 0.05. New infarct comprising 4% of LV mass is associated with adverse prognosis in patients with revascularization-related injury (21).

## Results

### Patients

In the CMR substudy, 205 consented to participate. Of these, 2 patients were excluded: 1 patient did not complete the early CMR, and in 1 patient, the LGE images were not analyzable. The complete revascularization and IRA-only groups in the CMR substudy were well-matched for characteristics, with no statistically significant differences between groups, although there was a trend for more women in the IRA-only group (Table 1).

### Angiographic and PCI details

Data are shown in Table 2. Thirty patients in the complete revascularization group had a staged procedure 1.43 (interquartile range [IQR]: 1.03 to 2.04) days after the PPCI. Coronary artery disease severity was similar in the groups, although the IRA-territory collateralization grade was significantly higher in the complete revascularization group. Total screening time, contrast dose, procedure length, and number of implanted stents were significantly greater in complete revascularization patients. The vast majority of patients in both arms received drug-eluting stents, although this was slightly higher in complete revascularization patients. Symptom-to-PCI times, antiplatelet, anticoagulant use, and post-PPCI creatine kinase rise were similar in both arms. There was a nonsignificant trend for no-reflow to be more common in the complete revascularization than the IRA group. There was greater usage of a second antianginal agent in patients in the IRA-only group.

**Table 2:** Periprocedural Details in the CR and IRA-Only Groups

	CR (n = 98)	IRA (n = 105)	p Value
Radial access	81/97 (83.5)	82/105 (78.1)	0.33
Symptom to PCI time, min∗	192 (131–302)	172 (127–268)	0.20
Glycoprotein IIb/IIIa inhibitor	34/97 (35.1)	36/104 (34.6)	0.95
Bivalirudin	52/92 (56.5)	43/94 (45.7)	0.14
Thrombectomy catheter	67/97 (69.1)	79/105 (75.2)	0.33
Contrast dose, ml∗	300 (220–400)	190 (150–230)	**<0.001**
Screening time, min∗	17 (12–23)	9 (7–13)	**<0.001**
Procedure length, min∗	66 (43–84)	42 (30–55)	**<0.001**
Vessels with ≥70% stenosis	1.8 ± 0.6	1.7 ± 0.6	0.82
Left anterior descending IRA	34/98 (34.7)	39/105 (37.1)	0.82
Left circumflex artery IRA	20/98 (20.4)	18/105 (17.1)	0.55
Right coronary artery IRA	44/98 (44.9)	48/105 (45.7)	0.91
Rentrop grade			
0–1	88/98 (89.8)	102/105 (97.1)	
2–3	10/98 (10.2)	3/105 (2.9)	**0.033**
TIMI pre-PCI grade	0 (0–1)	0 (0–1)	0.56
TIMI grade post-PCI	3 (3–3)	3 (3–3)	0.31
IRA no-reflow	8/98 (8.2)	3/107 (2.8)	0.09
Total number of stents	3 (2–4)	1 (1–2)	**<0.001**
Drug-eluting stent use	97/98 (99)	96/105 (91.4)	**0.013**
Aspirin	97/98 (99.0)	105/105 (100)	0.30
Second antiplatelet agent	98/98 (100)	105/105 (100)	1.00
Clopidogrel	34/98 (34.7)	36/105 (34.3)	0.95
Prasugrel	49/98 (50.0)	53/104 (51.0)	0.89
Ticagrelor	15/98 (15.3)	16/105 (14.3)	0.91
Beta-blocker	93/98 (94.9)	97/105 (92.4)	0.46
ACEI or ARB	95/98 (96.9)	101/105 (96.2)	0.77
Additional antianginal medication	6/98 (6.1)	17/105 (16.2)	**0.024**
Statin	98/98 (100)	104/105 (99.1)	0.33
Loop diuretic	9/98 (9.2)	13/105 (12.4)	0.46
Aldosterone inhibitor	5/98 (5.1)	5/105 (4.8)	0.91

Values are n/N (%), median (interquartile range), or mean ± SD. The **bold** type indicates statistically significant p values.

Additional antianginal medication includes calcium-channel blockers, nitrates, or nicorandil.

ACEI = angiotensin-converting enzyme inhibitor; ARB = angiotensin receptor blocker; TIMI = Thrombolysis In Myocardial Infarction; other abbreviations as in Table 1.

∗ Non-normally distributed data: analyzed after log transformation with independent Student *t* testing.

### Pre-discharge CMR

Results are displayed in Table 3. Pre-discharge CMR was undertaken at a median of 3 days post-PPCI in both treatment arms. There was no statistical difference in the primary endpoint of total infarct size between the groups: IRA-only, 13.5% (IQR: 6.2% to 21.9%) of LV mass versus complete revascularization, 12.6% (IQR: 7.2% to 22.6%) of LV mass, p = 0.57. The ratio of the geometric means for total infarct size in the IRA-only (15.9 ± 13.2%) and CR (16.3 ± 13.0%) arms is 0.98 (95% confidence interval: 0.82 to 1.41), confirming no difference between the 2 treatment arms. When corrected for covariates (age, sex, anterior MI, time to revascularization, TIMI flow pre-PCI, diabetic status, Rentrop grade and AAR), there remained no difference in median infarct size (beta = 0.02, p = 0.68) between the 2 groups.

**Table 3:** Pre-Discharge and Follow-Up CMR

Pre-Discharge CMR	CR (n = 98)	IRA (n = 105)	p Value
Total infarct size, % LV mass∗	12.6 (7.2–22.6), 16.3 ± 13.0	13.5 (6.2–21.9), 15.9 ± 13.2	**0.57**
Time from PPCI, days∗	3.0 (2.0–4.3)	2.8 (1.8–3.4)	0.13
Infarct on LGE	95 (96.9)	95 (90.5)	0.06
Patients with >1 infarct	22 (22.4)	11 (10.5)	**0.02**
Patients >1 acute infarct	17 (17.1)	5 (4.8)	**0.004**
Number of acute infarcts in those with >1 infarct†	2 (2–2), 2.2 ± 0.4	2 (2–2) 2.0 ± 0.0	0.60
IRA infarct size, % LV mass∗	12.1 (7.0–21.4), 15.2 ± 12.1	12.2 (6.2–21.2), 15.3 ± 13.2	0.68
Total acute IS, % LV mass∗	12.5 (7.0–22.0) 15.8 ± 12.4	12.4 (6.2–21.6) 15.4 ± 13.2	0.60
Acute NIRA infarct size, % LV mass in those with >1 infarct†	2.5 (0.54–4.5), 3.2 ± 3.3	2.1 (0.81–4.5), 2.5 ± 1.9	**0.004**
Acute NIRA infarct size (% LV mass, per infarct†)	1.4 (0.3–2.3), 1.6 ± 1.5	1.0 (0.4–2.2), 1.3 ± 1.0	0.94
Area at risk, % LV mass§	32.2 ± 11.8	36.0 ± 12.9	0.06
MSI§	58.5 (32.8–74.9)	60.5 (40.6–81.9)	0.14
MVO present	57/98 (58.2)	54/105 (51.4)	0.34
MVO, % LV mass†	0.19 (0.00–2.00)	0.08 (0.00–1.05)	0.63
IMH present§	22/75 (29.3)	17/77 (22.1)	0.31
RV infarction	7/98 (7.1)	4/105 (3.8)	0.29
LVMI, g/m^2^∗	52.3 (46.8–62.0)	52.2 (44.7–59.2)	0.33
LVEDVI, ml/m^2^∗	89.7 (80.7–102)	90.7 (80.4–102)	0.64
LVESVI, ml/m^2^∗	47.0 (38.0–58.4)	49.8 (39.7–62.1)	0.56
LVEF, %	45.9 ± 9.9	45.1 ± 9.5	0.60

Follow-Up CMR	(n = 84)	(n = 80)	
Time to CMR, months	9.4 (9.0–10)	9.3 (8.9–9.9)	0.20
LVMI, g/m^2^∗	47.4 (40–52.6)	43.4 (38.0–49.3)	0.33
LVEDVI, ml/m^2^∗	93.3 (82.2–110)	95.0 (82.7–107)	0.63
LVESVI, ml/m^2^∗	45.1 (37.8–58)	43.6 (34.8–57.9)	0.33
LVEF, %	49.7 ± 9.4	50.8 ± 8.7	0.42
Infarct on LGE	82/84 (97.6)	71/80 (88.8)	**0.023**
Patients with >1 infarct	20/84 (23.8)	9/80 (11.2)	**0.035**
IS, % LV mass∗	7.3 (3.0–14.4)	7.6 (3.2–15.1)	0.41
Final MSI∗	82.1 (63.0–90.3)	79.4 (71.6–93.3)	0.20

Perfusion	(n = 82)	(n = 77)	
Ischemic burden, %‡	3.4 ± 8.9	4.3 ± 11.3	0.81
Ischemia present	17/82 (20.7)	16/77 (20.8)	0.99
Ischemic burden in patients with ischemia, %	15.5 ± 13.7	20.4 ± 17.1	0.37
Ischemic burden >20%	6 (7.3)	6 (7.8)	0.91

Values are n/N (%), median (interquartile range), mean ± SD, or n (%), unless otherwise noted. The **bold** type indicates statistically significant p values.

IMH = intramyocardial hemorrhage; IS = infarct size; LGE = late gadolinium enhancement; LV = left ventricular; LVEDVI = left ventricular end-diastolic volume index; LVEF = left ventricular ejection fraction; LVESVI = left ventricular end-systolic volume index; LVMI = left ventricular mass index; MSI = myocardial salvage index; MVO = microvascular obstruction; NIRA = non–infarct-related artery; PPCI = primary percutaneous coronary intervention; RV = right ventricular; other abbreviations as in Table 1.

∗ Nonnormally distributed data: analyzed after log transformation with independent Student *t* testing.

† Nonnormally distributed data: analyzed using Mann-Whitney analysis.

‡ Because the median and interquartile range was 0 (0 to 0) for both IRA and CR groups, mean ± SD of the results are presented although the data are nonparametrically distributed.

§ Analyzable edema imaging available in 75 of the complete revascularization group and 77 of the IRA-only group.

**Figure 3:**
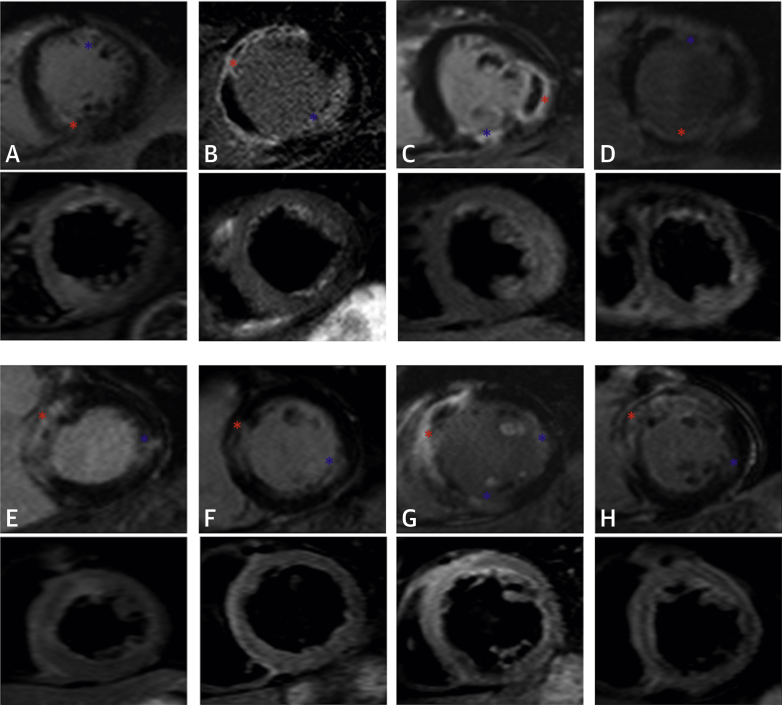
Examples of Patients With >1 “Acute” MI on CMR Late gadolinium-enhanced short-axis images (**top row and third rows**) and corresponding colocalized edema images **(second and fourth rows)**. **Red asterisks** indicate IRA-territory infarct; **blue asterisks** indicate NIRA-territory infarct(s). Subject ID: **(A)** (X511); **(B)** (X612); **(C)** (X665); **(D)** (X709); **(E)** (X757); **(F)** (X791); **(G)** (X798); **(H)** (X808). IRA infarct size and non-IRA PCI are shown in Online Table 1. NIRA = non–infarct-related artery; other abbreviations as in Figure 2.

The prevalence of multiple territory infarcts in the complete revascularization group was double that of the IRA-only group and the number of acute non-IRA infarcts was increased 3-fold in those undergoing complete revascularization (Table 3). Examples, with corresponding edema images, are shown in Figure 3, and the location, size of infarct, expected coronary artery territory, and whether the individual patients had an additional non-IRA PCI are shown in Online Table 1. Eighteen of 20 acute non-IRA infarcts in patents in the complete revascularization group concurred with additional PCI in the relevant non-IRA coronary territory. Five patients randomized to the IRA-only group also had non-IRA acute MI. Two of these patients had treatment crossover and received non-IRA PCI. The first crossover followed ongoing ischemia post-PPCI and was associated with non-IRA MI in the relevant territory. The second crossover resulted from human error, and this patient had a small non-IRA acute MI in the anteroseptum but had non-IRA PCI of the circumflex artery. Six patients in the IRA-only and 5 in the complete revascularization group had chronic infarcts (evidenced by wall thinning). Excluding these patients from the analysis did not affect the results (Online Table 2).

MVO was present in more than one-half of all patients, although quantitatively, the amount was very low (median <0.2% of LV mass). In 52 patients (26%), AAR could not be quantified: no artifact, but no edema discernable (n = 33); not performed due to arrhythmia or suboptimal breath-holding (n = 14); or severe artifact (n = 5). AAR and MSI were lower, but not significantly, in the complete revascularization group. LV volume, mass, and ejection fraction were similar in both groups.

### Follow-up CMR

Follow-up CMR was completed in 84 patients in the complete revascularization group and 80 patients in the IRA-only group (Table 3). Of the 39 patients who did not have a repeat CMR, 29 patients declined, 3 had died, 2 cited claustrophobia, 1 had an implantable cardioverter-defibrillator, 1 had a severe noncardiovascular illness, and in 3, there were logistical reasons. There were no differences in baseline characteristics or pre-discharge CMR between those who completed and did not attend the follow-up CMR (data not shown). Three patients were unable to undertake adenosine stress perfusion due to obstructive airways disease, and perfusion imaging was unanalyzable in 2 patients due to severe persistent dark-rim artefact (1 in the complete revascularization group, 1 in the IRA-only group). LV volumes and function were similar between groups. The prevalence of infarct and multiple infarcts were greater in the complete revascularization group. However, there was no significant difference in total infarct size and final MSI between the groups. Reversible perfusion defects were seen in 21% of patients in both groups, and overall ischemic burden was small. When the extent of ischemia was assessed only in patients with reversible perfusion defects, the ischemic burden was not statistically different in the complete revascularization and IRA-only groups.

### Clinical outcomes

Median follow-up was 372 days (IRA 377 days, complete revascularization 366 days, p = 0.38). One hundred ninety-eight (98%) patients attended 12-month clinical follow-up (3 patients died before this time point, and 2 patients withdrew consent). Length of inpatient stay and incidence of in-hospital clinical events were similar in the treatment arms (Table 4). There was a borderline significant reduction in MACE in patients undergoing complete revascularization, and the corresponding events rates and hazard ratio were similar to that seen in the main trial. Thirteen patients in the IRA-only arm had 14 revascularization procedures (2 separate PCIs in 1 patient) after PPCI. All but 1 were revascularization to the non-IRAs (1 patient had acute stent thrombosis of the IRA on day 0 and had repeat PCI). The indications were as follows: acute coronary syndrome 7 (3 non-STEMI); 6 refractory symptoms (1 coronary artery bypass surgery); and 1 patient underwent elective PCI at the discretion of the responsible physician.

**Table 4:** Clinical Outcomes

12-Month Follow-Up	CR (n = 98)	IRA (n = 105)	HR (95% CI)	p Value
MACE	8 (8.2)	18 (17.1)	0.43 (0.18–1.04)	0.055
Death	1 (1.0)	1 (1.0)	1.07 (0.07–17.4)	0.96
Recurrent MI	0 (0.0)	3 (2.9)	—	0.10
Heart failure	3 (3.1)	4 (3.8)	0.80 (0.17–3.7)	0.77
Revascularization	4 (4.1)	10 (9.5)	0.40 (0.12–1.3)	0.13

Inpatient Clinical Events			OR (95% CI)	
Length of inpatient stay, days	3 (2–4), 3.5 ± 2.6	3 (2–4), 3.9 ± 2.8		0.13
Death	1 (1.0)	1 (0.9)	1.07 (0.07–17.4)	0.96
Recurrent MI	0 (0.0)	1 (0.9)	2.17 (0.19–24.3)	0.33
Heart failure	2 (2.0)	1 (1.0)	0.71 (0.12–4.3)	0.52
Repeat revascularization	2 (2.0)	3 (2.9)	—	0.71
Safety endpoints				
Contrast nephropathy	1 (1.0)	0 (0.0)	—	0.30
Vascular access injury needing repair	0 (0.0)	0 (0.0)	—	1.00
CVA/TIA	0 (0.0)	0 (0.0)	—	1.00
Major bleed	3 (3.1)	1 (1.0)	3.29 (0.34–32.1)	0.28

Values are n (%), median (interquartile range), or mean ± SD.

A dash indicates that no HR was presentable because 1 or both treatment arms had an incidence of 0.

CI = confidence interval; CVA = cerebrovascular accident; HF = heart failure; HR = hazard ratio; MACE = major adverse cardiovascular events; OR = odds ratio; TIA = transient ischemic attack; other abbreviations as in Table 1.

## Discussion

This is the first detailed study of pre-discharge and follow-up CMR outcomes in a randomized study of IRA-only versus complete revascularization in multivessel coronary disease at PPCI. The data have confirmed that non-IRA PCI is associated with additional infarction. However, these type 4a MIs (6) are relatively infrequent, generally small, and did not result in an increase in total infarct size. There is mounting evidence from randomized trials that treating multivessel disease with complete revascularization 4, 22 leads to a reduction in MACE after PPCI compared with an IRA-only strategy.

The patients in the substudy had similar baseline characteristics to those in the main trial. Because time to revascularization (4) and anterior MI (23) are strongly associated with infarct size, randomization was stratified by these variables. There was a similar reduction in the hazard ratio for MACE in the complete revascularization CMR subgroup as that seen in the main study compared with IRA-only revascularization, and we believe that the CMR substudy population is representative of those in the main study.

It is well-recognized that elective PCI can cause a troponin rise in approximately 30% of patients and approximately 50% undergoing PCI for unstable angina (24). Such type 4a MIs (6) can be detected on CMR and have been associated with adverse prognosis 21, 25. In this substudy of CvLPRIT, the prevalence of >1 CMR-detected infarct in patients receiving complete revascularization was double that in the IRA-only arm (23.8% vs. 11.2%), and more than 3-fold for the acute non-IRA infarcts (17.1% vs. 4.8%) (Central Illustration). Previous Q-wave MI was an exclusion criterion in this study, but 4% had a history of previous non-STEMI, and a similar number (6% in the IRA-only and 5% in the complete revascularization groups) had chronic non-IRA MI on the pre-discharge CMR. Excluding these patients did not significantly affect the results. These data suggest that an additional 12% of patients with multivessel disease who receive complete revascularization at the time of PPCI will have evidence of additional CMR-detectable infarction compared with IRA-only revascularization. However, this proportion is less than might have been expected from previous studies in elective PCI (24), where up to 29% of patients have evidence of new infarction on CMR associated with troponin elevation (25). The extent of acute non-IRA infarction was also smaller (median 2.5% of LV mass) than may have been anticipated from elective PCI data given that average infarct size in those with new late enhancement on CMR was 5.0 ± 4.8% of LV mass (25), despite all patients in that study being pre-treated with clopidogrel for >24 h and given a glycoprotein IIb/IIIa inhibitor periprocedurally. Importantly, in the present study, total infarct size was not increased in the short term or at follow-up, and there were no significant differences in myocardial salvage, LV volumes, or ejection fraction between the treatment groups. Peak creatine kinase levels were also similar in the 2 groups.

**Central Illustration:**
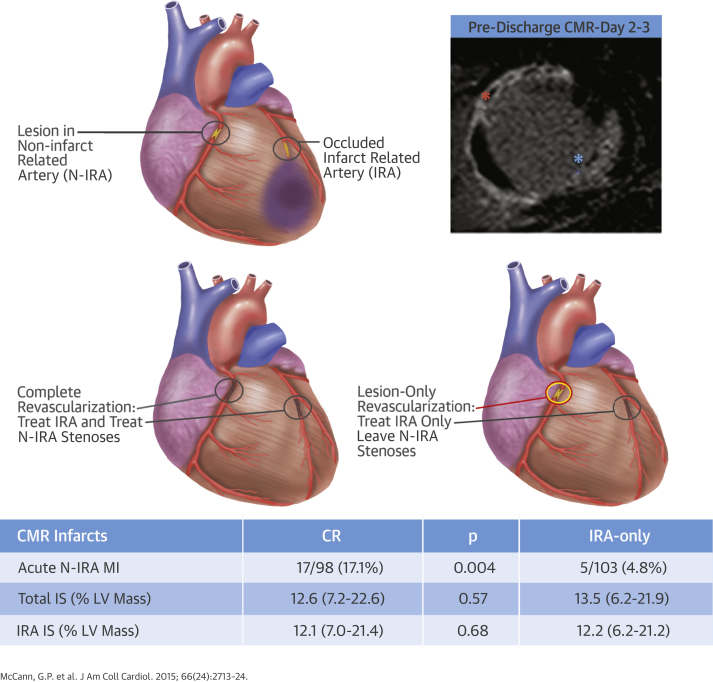
Complete Versus Lesion-Only Revascularization in Acute MI: The CMR CvLPRIT Substudy Overview of the CvLPRIT CMR trial showing the randomization strategy and main results. **Red asterisk** indicates IRA late gadolinium enhancement; **blue asterisk** indicates N-IRA late gadolinium enhancement. CMR = cardiovascular magnetic resonance; CvLPRIT = Complete Versus Lesion-Only Primary PCI Pilot Study; LV = left ventricular; MI = myocardial infarction; N-IRA = non–infarct-related artery; IS = infarct size.

These findings provide reassurance that non-IRA intervention at the time of PPCI does not lead to increased total infarct size. In the main CvLPRIT trial, complete revascularization resulted in a significantly reduced hazard ratio for 12-month MACE despite the greater prevalence of CMR-detected type 4a MIs shown here. There are limited data as to whether revascularization-induced myocardial injury detected by CMR is linked to prognosis (21), and none in patients presenting with STEMI. In an observational study of 152 patients undergoing elective revascularization, 32% had evidence of new LGE, which averaged 5 g (4% of LV mass), and one-half of these patients were treated with coronary artery bypass surgery (21). In that study, patients with new infarction following revascularization had reduced ejection fraction, increased LV volume, increased total infarct size, and a 3-fold increase in MACE at a median of 2.9 years follow-up compared with those without new LGE (21). Given that the complete revascularization group in the current study had no increase in total infarct size, LV volume, or reduced ejection fraction, it seems unlikely that the short- to medium-term clinical benefits of complete revascularization (22) will be offset in the long term by increased heart failure or sudden cardiac deaths. However, longer-term follow-up of patients in this study is needed to confirm this.

We did not observe any significant differences in myocardial salvage between the treatment groups in this study. Non-IRA revascularization at the time of PPCI could increase perfusion to watershed areas by relieving flow-limiting stenoses, resulting in increased myocardial salvage (26). Alternatively, resting myocardial perfusion and flow reserve following PCI may actually be reduced, as has been shown in elective patients as a result of distal embolization, particularly when the PCI is associated with new LGE 26, 27. It may be that both effects are seen with non-IRA PCI resulting in no net benefit with regard to myocardial salvage in the PPCI setting.

Unexpectedly, we also observed no difference in ischemic burden between the groups undergoing follow-up stress perfusion CMR. There are several potential explanations for this finding. First, it is well recognized that even severe angiographic stenoses may not cause ischemia 28, 29. Second, 11 patients in the IRA-only arm had further PCI before the stress CMR that is likely to have reduced ischemic burden in this group. Third, the small number of crossovers from randomization is likely to have diminished the differences in ischemia between the groups. Finally, the stress CMR was undertaken in patients on optimal medical therapy, which may dramatically reduce post-MI ischemia (30) making it more difficult to detect differences between the groups, especially as there was higher use of a second antianginal medication in the IRA-only group. This may also explain why the overall ischemic burden in our study was small (3% to 4%). It remains to be determined whether ischemia is prognostically important in the PPCI era, especially because medical therapy may result in similar clinical outcomes to a revascularization strategy even in patients treated with thrombolysis (30). Further insight on this subject will be available from the CvLPRIT nuclear substudy.

### Study limitations

The optimal timing to assess infarct size post-STEMI is uncertain (31). We chose an early time point to enhance participation in the CMR substudy because we felt there could have been a higher dropout rate scanning patients after hospital discharge. MSI was only reliably measured in ∼75% of patients, and the use of novel T1 or T2 mapping techniques for future studies may lead to a more robust assessment. Current CMR techniques cannot reliably differentiate whether a very small MI, which is not associated with wall thinning, edema, or MVO, is acute or chronic, and this contributed to the slight overreporting of acute non-IRA MIs that were not associated with revascularization in this study.

## Conclusions

An in-hospital complete revascularization strategy in patients with multivessel disease at the time of PPCI is associated with a small increase in type 4a MIs in non-IRA territories, but total infarct size was not significantly different compared with an IRA-only strategy.

Perspectives **COMPETENCY IN MEDICAL KNOWLEDGE:** In patients with STEMI and multivessel disease, a strategy of complete revascularization is associated with a small increase (12%) in the risk of type 4a MIs, but similar total infarct size, compared with a strategy addressing only the infarct-related artery.

**TRANSLATIONAL OUTLOOK:** Future trials should incorporate selective revascularization strategies based on coronary angiography at the time of primary PCI and functional assessments of coronary lesions to guide complete revascularization.
