# Supplementary material for: Complete Versus Lesion-Only Primary PCI: The Randomized Cardiovascular MR CvLPRIT Substudy
Source: J Am Coll Cardiol. 2015 Dec 22;66(24):2713–24. doi: 10.1016/j.jacc.2015.09.099 (PMC4681843; doi:10.1016/j.jacc.2015.09.099)
Supplement: Online Appendix and Online Tables 1 and 2 [file mmc1.docx]

**Supplemental data**

**Intra- and inter-observer variability**

Intraclass correlation coefficient for intraobserver agreement was 0.996, 0.988, 0.976, 0.995, 0.991 (for LVEDVI, LVESVI, LVEF, AAR, IS respectively) and for interobserver 0.995, 0.996, 0.996, 0.976, 0.922 (for LVEDVI, LVESVI, LVEF, AAR, IS respectively).

**Philips Sequence parameters**

Philips Intera 1.5T scanner

*T2w-STIR (area at risk):* 10mm slice thickness, no gap, matrix 208-256 x 256, FOV ~300-360 x 360-420, echo train length (20-40), coil signal intensity correction on, TR 2.2s, TE 60ms, flip angle 90^o^

*Late gadolinium enhancement:* 8mm slice thickness, 2mm gap, matrix 208-256 x 256, FOV 300 x 400, TI 220-360ms (progressive optimization), TR 4.5ms, TE 1.8ms, flip angle 15^o^

**Supplemental Table 1: Patients with 2 or more ‘acute’ MI: Infarct locations, size and relationship to additional non-IRA PCI**.

| **ID** | **No. of infarcts** | **IRA** | **MI area** | **IRA IS % LV** | **MI 2**  **Area** | **IS 2 % LV** | **IS 3 Area** | **IS 3 %LV** | **CMR**  **N-IRA** | **Actual N-IRA PCI** | **Timing of**  **N-IRA PCI** |
| --- | --- | --- | --- | --- | --- | --- | --- | --- | --- | --- | --- |
| **Complete revascularization patients** | | | | | | | | | | |  |
| X511 | 2 | RCA | I | 19.09 | Apical | 3.82 | -- | -- | LAD | LAD | Index |
| X517 | 2 | LAD | AS | 13.70 | AL | 0.93 | -- | -- | Diagonal | Diagonal | Staged |
| X530 | 2 | RCA | I | 25.94 | AS | 0.06 | -- | -- | LAD | LAD | Staged |
| X540 | 2 | RCA | I | 9.34 | L | 2.82 | -- | -- | LCX | LAD **^§^** | Staged |
| X545 | 2 | LAD | AS | 1.56 | IL | 5.19 | -- | -- | LCX | LCX | Staged |
| X594 | 3 | RCA | I | 4.27 | AL | 0.94 | A | 0.10 | LCX +LAD | LCX +LAD | Index |
| X599 | 3 | LCX | L | 9.82 | I | 8.93 | AS | 0.98 | RCA + LAD | RCA + LAD | Index |
| X612 | 2 | LAD | A | 42.20 | I | 4.37 | -- | -- | RCA | RCA | Index |
| X665 | 2 | LCX | L | 24.13 | I | 4.35 | -- | -- | RCA ^¶^ | LAD | Index |
| X695 | 2 | RCA | I | 7.75 | AS | 5.01 | -- | -- | LAD | LAD | Index |
| X747 | 2 | LAD | AS | 22.12 | I | 0.37 | -- | -- | RCA | RCA | Index |
| X757 | 2 | LAD | AS | 20.83 | L | 0.60 | -- | -- | LCX | LCX | Staged |
| X785 | 2 | RCA | IL | 37.45 | AS | 0.15 | -- | -- | LAD | LAD | Staged |
| X788 | 2 | LAD | AS | 4.73 | IL | 2.08 | -- | -- | LCX | LCX | Index |
| X791 | 2 | LAD | AS | 7.06* | IL | 11.86 | -- | -- | LCX | LCX | Staged |
| X798 | 3 | LAD | AS | 34.87 | I | 0.66 | L | 2.03 | RCA +LCX | RCA +LCX | Staged |
| X808 | 2 | LAD | AS | 49.30 | IL | 2.85 | -- | -- | LCX | LCX | Staged |
| **IRA only PCI patients** | | | | | | | | | | |  |
| X661 | 2 | LAD | AS | 10.56 | I | 0.11 | -- | -- | -- | -- |  |
| X709 | 2 | LCX | IL | 21.77 | Apical | 2.10 | -- | -- | LAD | LAD | Index |
| X716 | 2 | RCA | I | 11.25 | AS | 4.54 | -- | -- | LAD | Cx | Index |
| X719 | 2 | LAD | AS | 21.08 | IL | 4.40 | -- | -- | -- | -- |  |
| X728 | 2 | LAD | AS | 24.86 | I | 1.50 | -- | -- | -- | -- |  |
|  |  |  |  |  |  |  |  |  |  |  |  |

*Abbreviations: AL=Anterolateral; AS=Anteroseptal; I=inferior; IL= Inferolateral; Timing of N-IRA PCI: Index-performed at same sitting as PPCI: Staged: deferred at operator’s discretion to delayed inpatient N-IRA PCI.*

***^§^*** *LAD PCI crossed Diagonal; ^¶^ Co-dominant system.*

**Supplemental Table 2: CMR data excluding patients with chronic infarcts on the acute scan**

| **Variable** | **CR** | **IRA** | **p** |
| --- | --- | --- | --- |
| **Acute CMR** | **n=93** | **n=99** |  |
| **Total IS (% LVM)**  **Median (IQ range)**  **Mean±SD** | **12.5 (7.0-21.3)**  **[15.5±12.3]** | **12.6 (4.8-22.0)**  **[15.7±13.6]** | **0.48** |
| Time from PPCI (days) | 3.0 (2.0-4.2) | 2.8 (1.9-3.5) | 0.19 |
| Infarct on LGE (%) | 90 (96.8) | 89 (89.9) | 0.06 |
| Patients with >1 infarct (%) | 17 (18.3) | 5 (5.1) | **0.004** |
| IRA Infarct size (% LV Mass)  Median (IQ range)  Mean±SD | 12.1 (7.0-20.6)  14.9±12.0 | 12.4 (4.8-21.8)  15.6±13.5 | 0.79 |
| **Follow-up CMR** | **n=79** | **n=76** |  |
| Time to CMR (months) | 9.4 (8.9-10.0) | 9.3 (9.0-10.0) | 0.70 |
| Infarct on LGE (n,%) | 77/79 (97.5) | 67/76 (88.8) | **0.024** |
| Patients with >1 infarct (%) | 15/79 (19.0) | 5/76 (6.6) | **0.021** |
| IS (% LVM) | 7.0 (3.0-13.5) | 7.4 (3.0-14.7) | 0.37 |
|  |  |  |  |
| **Perfusion** | **n=77** | **n=73** |  |
| Ischaemic burden (%)* | 3.0±7.6 | 3.7±9.7 | 0.64 |
| Ischaemia present (%) | 15/77 (19.5) | 15/73 (20.5) | 0.87 |
| Ischaemic burden (%) in patients with ischaemia | 14.4±11.3 | 17.9±14.6 | 0.46 |
| Ischemic burden > 20% | 5 (6.5%) | 5 (6.8%) | 0.93 |

Acute IS corrected (age, anterior MI, TIMI pre, TTR, sex, DM, AAR, Rentrop) gives a p-value of 0.569 comparing IRA vs. CR
